# Analysis of Mercury Content in Medicinal Plants Popular in Poland

**DOI:** 10.3390/molecules31193569

**Published:** 2026-10-08

**Authors:** Barbara Brodziak-Dopierała, Agnieszka Fischer

**Affiliations:** Department of Toxicology, Toxicological Analysis and Bioanalysis, Faculty of Pharmaceutical Science, Medical University of Silesia, 8b Jedności Str., 41-200 Sosnowiec, Poland; afischer@sum.edu.pl

**Keywords:** mercury (Hg), wild populations, commercial products, medicinal plants, wild rose—*Rosa canina* L., common hawthorn—*Crataegus monogyna* L., dandelion—*Taraxacum officinale*, common nettle—*Urtica dioica* L., silver birch—*Betula pendula*

## Abstract

The use of herbal medicinal products and dietary supplements has increased significantly in recent years. Insufficient knowledge regarding the effects of herbal raw materials, as well as their potential adverse effects and interactions with medications and food, poses a considerable risk in therapies involving these products. Furthermore, contamination with heavy metals, including mercury, may constitute an additional health hazard. The aim of this study was to determine the mercury (Hg) content in wild-growing herbal raw materials collected independently and in commercial products, namely herbal teas derived from the same plant species under investigation. The measured Hg levels were compared with the limits established by the applicable standard set out in Commission Regulation (EU) 2018/73. For the study, five medicinal plant species commonly used in Poland were selected: wild rose (*Rosa canina* L.), common hawthorn (*Crataegus monogyna* L.), dandelion (*Taraxacum officinale*), common nettle (*Urtica dioica* L.) and silver birch (*Betula pendula*). The determination of Hg concentrations was carried out using an AMA 254 (Altec, Prague, Czech Republic) atomic absorption spectrometer. The arithmetic mean Hg concentration across all analyzed samples was 18.68 µg/kg, with values ranging from 0.19 to 202.83 µg/kg. The highest median Hg concentrations were found in common nettle (23.80 µg/kg), followed by dandelion (13.00 µg/kg) and silver birch (12.68 µg/kg). Considerably lower concentrations were observed in common hawthorn (5.91 µg/kg) and wild rose (0.61 µg/kg). Statistically significant differences in Hg content were detected among the studied plant species (*p* < 0.001). With regard to individual morphological plant parts, the highest accumulation of Hg was observed in roots, followed by herbs, leaves, and buds. Among all analyzed samples, 32% exceeded the permissible Hg limit, with the majority of these being self-collected samples. Self-collection of medicinal plant materials, including common nettle (*Urtica dioica*), dandelion (*Taraxacum officinale*), and silver birch (*Betula pendula*), may pose a risk associated with Hg contamination. Given the continuously increasing demand for and market value of herbal medicines, ensuring the safety, quality, and efficacy of medicinal plants and herbal products is of great importance. Therefore, regular monitoring of toxic element levels in plant materials used in phytotherapy is justified and essential.

## 1. Introduction

In recent years, interest in herbal medicines and dietary supplements has been growing steadily. The growing use of herbal products can be attributed to several factors, including the high prevalence of chronic diseases, increased concern for health and physical appearance, rising costs of conventional medicines, the search for alternative treatment options, and the public perception that herbal products are safer than synthetic drugs. Herbal medicine is becoming increasingly popular, and plant-based preparations are often used for health promotion and disease prevention without prior medical consultation [1,2,3].

The global medicinal herb market is estimated to reach USD 253.8 billion by 2026 and is expected to continue growing through 2035. More than 50,000 plant species are used for medicinal purposes worldwide. According to international health reports, approximately 80% of the global population relies on herbal remedies to meet basic healthcare needs. China and India together account for more than 60% of the world’s medicinal plant production [4]. Europe represents approximately 25% of the global market share, and the European nutraceutical sector is projected to grow from EUR 98 billion in 2024 to EUR 192.2 billion by 2034 [4]. The COVID-19 pandemic further increased public interest in herbal supplements, as consumers sought alternative products to support immune function [5]. Currently, adaptogens are among the most popular categories of herbal products.

It should also be noted that both herbal medicinal products and plant-based dietary supplements are available on the market, and that these two categories differ in their safety requirements and quality standards. Studies indicate that 63% of European consumers obtain information about herbal supplements from non-professional sources, such as social media platforms [5].

Despite their numerous beneficial properties, plant materials and herbal products used in the daily diet may contain harmful substances accumulated from the environment, such as heavy metals, including mercury (Hg), originating from soil, water, and air. Therefore, monitoring the quality and safety of plant-derived raw materials is essential to minimize potential health risks associated with their consumption [6,7,8].

Hg inhibits protein synthesis in plant leaves and disrupts several metabolic processes [9,10]. In particular, it adversely affects photosynthesis, chlorophyll production, gas exchange, and respiration, while also restricting seedling growth and root development [10]. Human exposure to Hg can likewise lead to numerous adverse health effects, including damage to the nervous system and kidneys [11,12].

Wild rose (*Rosa canina* L.) is a Rosaceae shrub whose fruits are rich in phenolic compounds, carotenoids, tocopherols, vitamin C, minerals, and galactolipids [13]. These constituents confer antioxidant, anti-inflammatory, immunomodulatory, cardioprotective, antidiabetic, and antibacterial activities. Galactolipids are particularly valued for their beneficial effects in osteoarthritis management [13,14,15,16].

Common hawthorn (*Crataegus monogyna* L.) is valued for its high content of polyphenols, flavonoids, phytosterols, and triterpenoid acids, which contribute to its strong antioxidant activity [17,18]. Hawthorn exhibits cardiotonic, hypotensive, diuretic, antispasmodic, and anti-atherosclerotic effects, and may also inhibit cancer cell proliferation and induce apoptosis [19,20,21,22,23,24].

Common nettle (*Urtica dioica* L.) contains flavonoids, tannins, vitamins, carotenoids, fatty acids, sterols, and phytoestrogens [25]. Owing to these compounds, it demonstrates antihistaminic, anti-inflammatory, antioxidant, diuretic, and hepatoprotective properties and is commonly used in the management of rheumatic disorders, kidney stones, and benign prostatic hyperplasia [25,26,27,28].

Dandelion (*Taraxacum officinale*) is rich in sesquiterpenes, triterpenes, phenolic acids, flavonoids, phytosterols, and inulin [29,30]. It supports liver function, exhibits choleretic activity, enhances immune responses, and may aid glycemic control and weight management [31].

Silver birch (*Betula pendula*) contains flavonoids, phenolic acids, triterpenes, tannins, and minerals that contribute to its anti-inflammatory, analgesic, and diuretic effects [32,33,34]. Traditionally used to support urinary tract health and relieve rheumatic conditions, birch also shows antioxidant, immunomodulatory, and potential anticancer properties [35,36,37].

According to available reports, Poland is one of the major emitters of mercury (Hg) to the atmosphere among the countries of the Baltic Sea region. At the same time, the reduction in Hg emissions has been less pronounced than in most other European countries. Analyses of living organisms inhabiting both terrestrial and aquatic environments have revealed moderate or low concentrations of total mercury (THg) compared with those reported from other regions of the world. This may be attributed to the insufficient implementation of an atmospheric mercury monitoring program in Poland [38].

The average concentration of total gaseous mercury (TGM) in ambient air was 1.65 ng·m^−3^ in 2024 and 1.52 ng·m^−3^ in 2025. Mercury concentrations in soil are related both to the geological characteristics of a given area and to anthropogenic activities. The average THg content in Polish soils generally did not exceed 50 ng·g^−1^ dry weight (d.w.), which is considered to represent the geochemical background level. In non-urbanized areas, the mean THg concentration in soil was 17 ng·g^−1^ d.w. In southern Poland, higher THg concentrations in soil, exceeding 60 ng·g^−1^ d.w., were associated with elevated mercury levels in the underlying bedrock [39].

Previous studies have demonstrated significant differences in Hg accumulation among plant organs. In most plant species, roots contain the highest mercury concentrations due to restricted root-to-shoot translocation, whereas leaves may accumulate substantial amounts of Hg through atmospheric deposition [40]. In medicinal plants, elevated Hg concentrations have frequently been reported in leaves, highlighting the importance of foliar uptake from polluted air. Leaves are a significant site of Hg accumulation, and its content increases with leaf age. The authors indicate that accumulation in above-ground organs may primarily reflect Hg uptake from the atmosphere [41].

The presence of Hg in the environment, increasing public awareness of the risks associated with toxic metal contamination, and the widespread use of self-collected herbal preparations prompted this study to determine Hg concentrations in selected medicinal plant materials and commercial products prepared from these plants. The study focused on plant species that are commonly found in Poland and can be easily collected by individuals: wild rose (fruits), common hawthorn (fruits and flowers), silver birch (buds and leaves), dandelion (roots, herbs, and flowers), and common nettle (roots and leaves). The conducted analyses aimed to assess whether self-collected plant materials are safe for consumption and to identify which plant species and/or morphological parts accumulate the highest amounts of Hg. The measured Hg concentrations in the selected plant materials and herbal infusions were compared with the maximum permissible level established by Commission Regulation (EU) 2018/73 [42], namely 0.02 µg/g (20 µg/kg).

## 2. Results and Discussion

The arithmetic mean Hg concentration across all analyzed samples was 18.68 µg/kg, while the median value was considerably lower at 13.14 µg/kg. The minimum and maximum concentrations were 0.19 µg/kg and 202.83 µg/kg, respectively. A high coefficient of variation (120%) was observed, indicating substantial variability in Hg concentrations among the analyzed plant samples. Among the five medicinal plant species examined, the highest median Hg concentration was found in common nettle (23.80 µg/kg), followed by dandelion (13.00 µg/kg) and silver birch (12.68 µg/kg). Considerably lower concentrations were determined in common hawthorn (5.91 µg/kg), while the lowest Hg levels were detected in wild rose (0.61 µg/kg). The coefficients of variation were also highest for the species with the greatest Hg concentrations, reaching 102% and 109% for common nettle and dandelion, respectively. For silver birch, hawthorn, and wild rose, the coefficients of variation were 74%, 89%, and 54%, respectively. Regarding the minimum and maximum Hg concentrations, the widest range was observed in common nettle (0.60–202.83 µg/kg), followed by dandelion (0.28–115.06 µg/kg), silver birch (1.26–53.68 µg/kg), common hawthorn (0.97–38.92 µg/kg), and wild rose (0.19–1.41 µg/kg) (Table 1).

Statistically significant differences in Hg concentrations were observed among the studied plant species (*p* < 0.001). Pairwise comparisons revealed that nettle differed significantly from all other plant species examined, including wild rose, hawthorn, silver birch, and dandelion (*p* < 0.001). Dandelion showed significant differences relative to wild rose and nettle (*p* < 0.001) and to hawthorn (*p* < 0.01). Likewise, silver birch differed significantly from wild rose and nettle (*p* < 0.001), as well as from hawthorn (*p* < 0.05). Hawthorn was significantly different from nettle (*p* < 0.001), dandelion (*p* < 0.01), and both wild rose and silver birch (*p* < 0.05).

When Hg concentrations were analyzed in individual morphological plant parts, roots proved to be the organs with the highest Hg accumulation. The mean Hg concentration reached 46.89 µg/kg in common nettle roots and 28.47 µg/kg in dandelion roots. High Hg concentrations were also observed in dandelion herbs, with a mean value of 27.78 µg/kg, and in common nettle leaves, where the mean concentration was 23.90 µg/kg. Mercury concentrations in silver birch leaves were of a similar magnitude, averaging 22.36 µg/kg. In contrast, Hg levels in birch buds were approximately 40% lower, with a mean concentration of 12.98 µg/kg. Hg concentrations below the overall mean value for all analyzed samples (18.68 µg/kg) were recorded in hawthorn flowers (8.00 µg/kg) and hawthorn fruits (8.49 µg/kg). The lowest Hg concentration was determined in wild rose fruits, with a mean value of 0.59 µg/kg (Table 2). Statistically significant differences in Hg concentrations were observed among the various morphological parts of the studied plants (*p* < 0.001).

For the median (Me) Hg concentrations in individual morphological plant parts, the highest value was observed in roots (23.84 µg/kg). Similar Hg concentrations were found in leaves and herbs, with median values of 19.04 µg/kg and 18.36 µg/kg, respectively, while slightly lower concentrations were detected in buds (12.11 µg/kg). Flowers (Me = 6.52 µg/kg) and fruits (Me = 1.77 µg/kg) exhibited the lowest Hg concentrations (Table 2).

**Figure 1:**
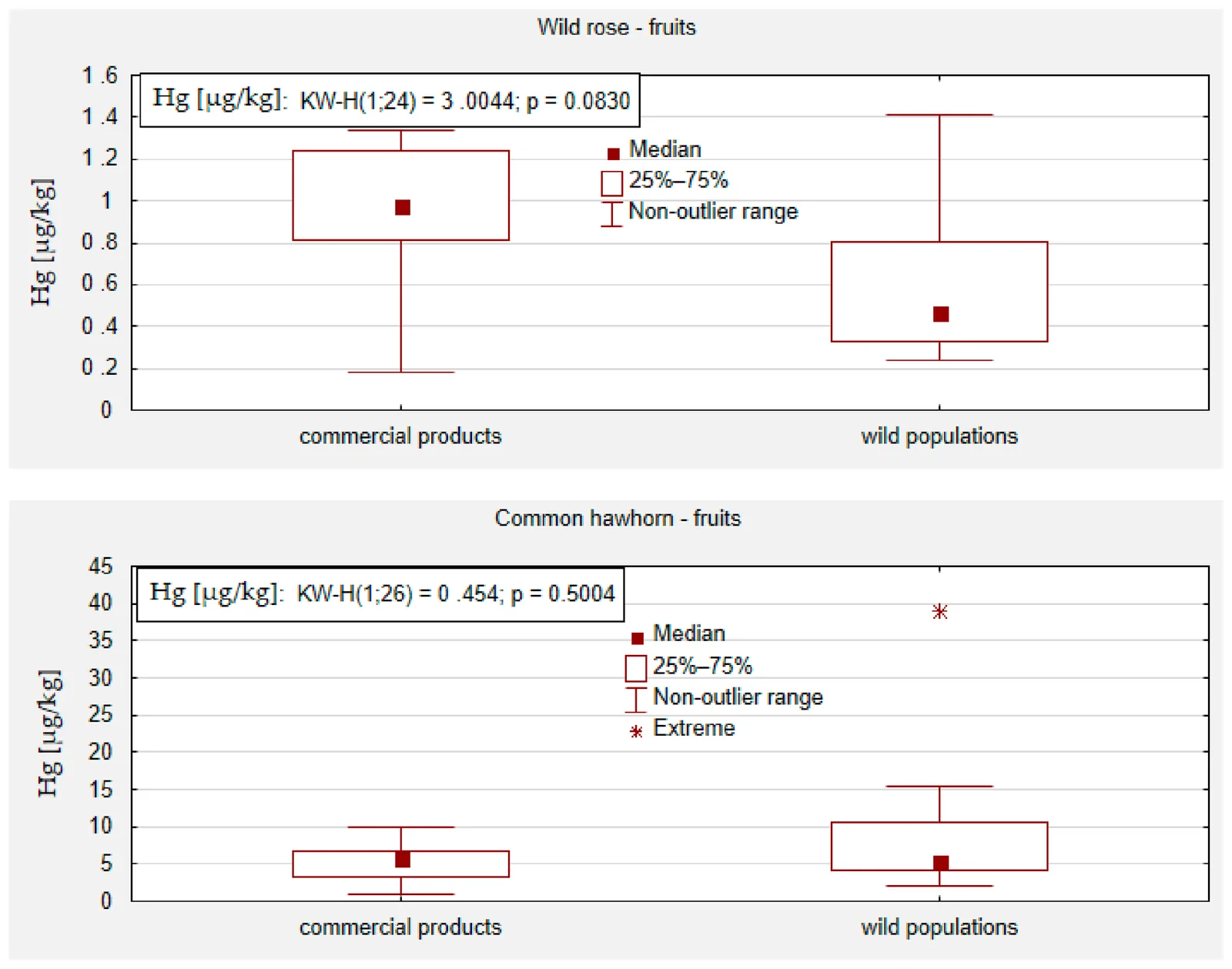
Comparison of Hg concentrations [µg/kg] in wild rose and hawthorn fruits.

Subsequently, Hg concentrations were compared between self-collected plant materials and commercial products derived from the same plant species. For four species, namely silver birch, common hawthorn, dandelion, and common nettle, Hg concentrations were higher in self-collected wild-growing plant materials than in commercial products. The respective Hg concentrations were 18.21 vs. 7.09 µg/kg for silver birch, 8.49 vs. 7.18 µg/kg for common hawthorn, 21.42 vs. 4.03 µg/kg for dandelion, and 35.40 vs. 10.88 µg/kg for common nettle. The largest differences were observed for dandelion, common nettle, and silver birch, and these differences were statistically significant (*p* < 0.001). No statistically significant differences were found between self-collected plant materials and commercial products in the case of common hawthorn and wild rose (Figure 1). Wild rose was the only species in which higher Hg concentrations were detected in commercial products (0.92 µg/kg) compared with self-collected fruits (0.59 µg/kg).

**Figure 2:**
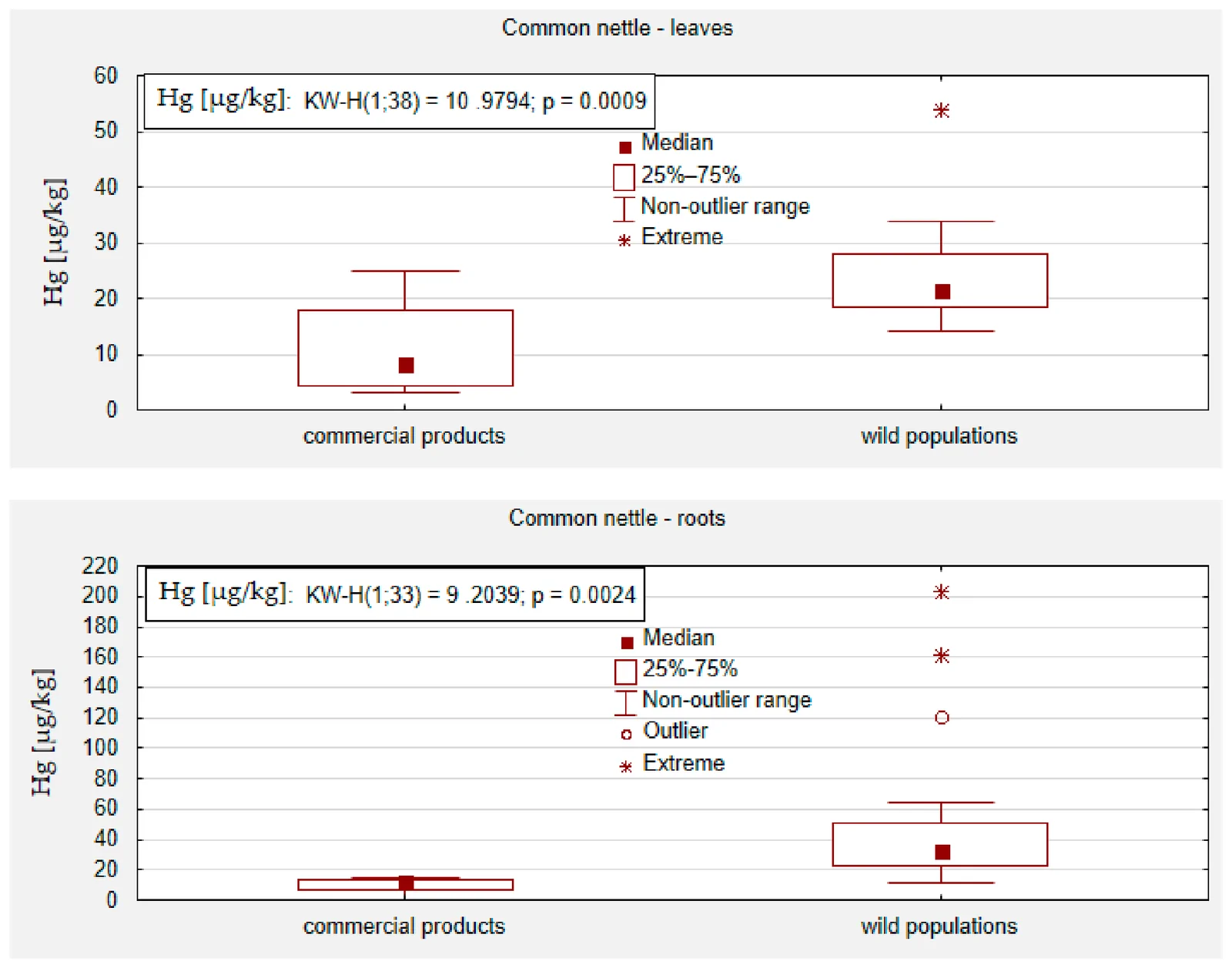
Comparison of Hg concentrations [µg/kg] in the leaves and roots of common nettle.

**Figure 3:**
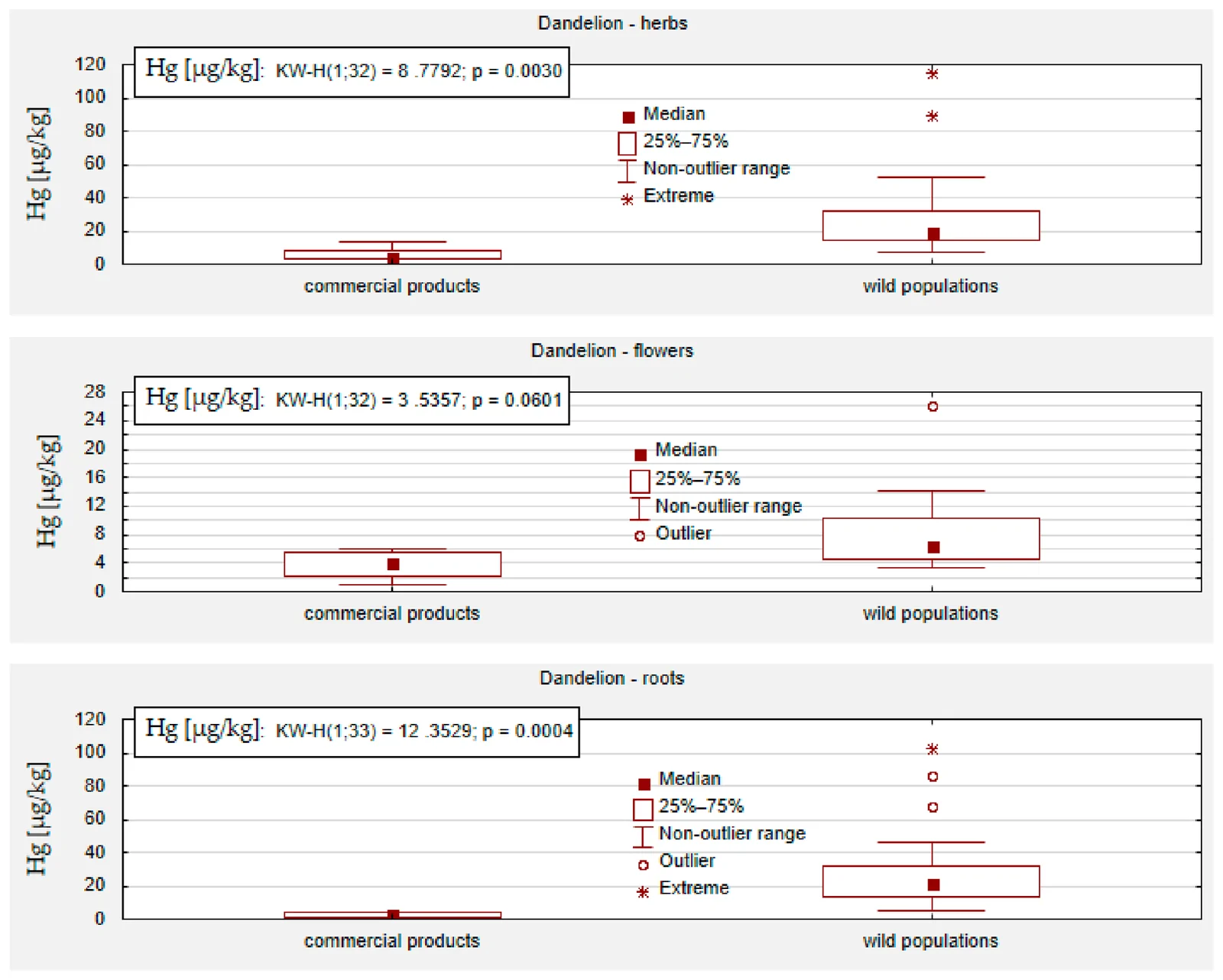
Comparison of Hg concentrations [µg/kg] in the roots, flowers, and herbs of dandelion.

**Figure 4:**
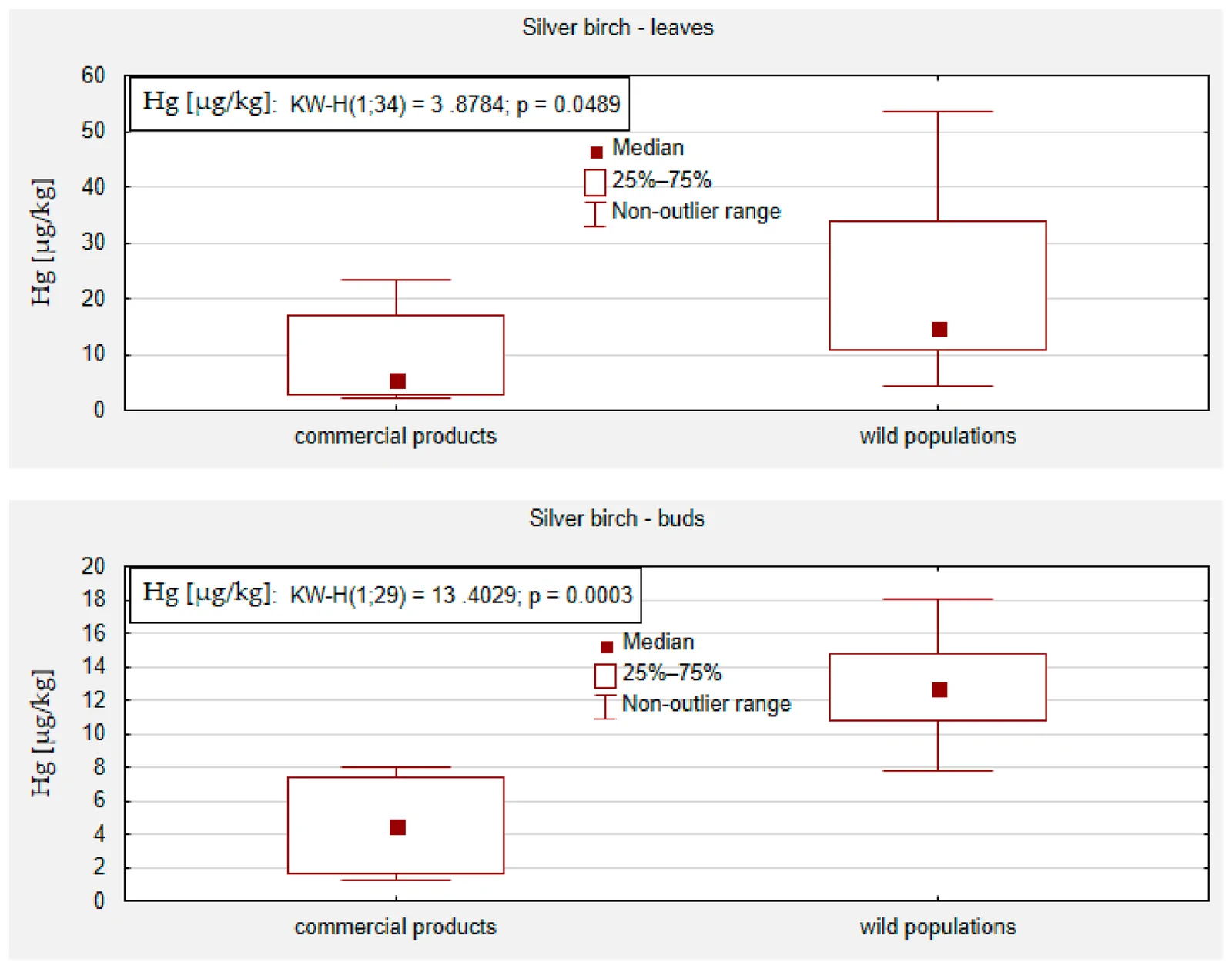
Comparison of Hg concentrations [µg/kg] in the leaves and buds of silver birch.

Analysis of individual plant species and their morphological parts revealed that, in nettle, both roots and leaves showed statistically significant differences (*p* < 0.005) between self-collected plant materials and commercial products (Figure 2). In dandelion, significant differences were observed for herbs (*p* < 0.025) and roots (*p* < 0.001), whereas no significant differences were found for flowers (Figure 3). In silver birch, statistically significant differences were detected only for buds (*p* < 0.001) (Figure 4).

When the obtained results were compared with the maximum permissible Hg level in herbal materials (20 µg/kg), it was found that 32% of the analyzed samples (N = 91) exceeded this limit. Among these, the vast majority were self-collected plant materials (N = 88), whereas only three samples were commercial products, including two samples of nettle leaves and one sample of birch leaves.

These findings indicate that more than one-third (38.4%) of the self-collected plant samples and over 5% of the analyzed commercial products exceeded the permissible Hg concentration. Exceedances were most frequently observed in common nettle (50% of samples), followed by dandelion (34%) and silver birch (15%). In the case of common hawthorn fruits, only a single sample exceeded the established limit.

With regard to plant morphological parts, exceedances of the permissible Hg level occurred predominantly in roots (49% of samples), followed by leaves (35%) and herbs (14%).

Consumers of plant-derived products often hold the misconception that “natural” automatically means “safe.” However, these terms are not synonymous. Consequently, regulatory policies governing herbal medicines and dietary supplements require standardization and continuous quality control [43]. Safety remains a major concern in the use of medicinal plants and necessitates the implementation of appropriate regulations to protect public health by ensuring product quality and consistency [43,44].

Herbal products used for therapeutic purposes require rigorous toxicological assessment, particularly with regard to contaminants absorbed by plants from the environments in which they grow. Without adequate quality control, plant-based therapies may cause adverse health effects instead of delivering their intended benefits. Plants are capable of accumulating metals from soil, water, and air. Furthermore, agricultural practices, storage conditions, and processing methods may introduce additional contaminants, including pesticides, pesticide metabolites, and synthetic fertilizers. Therefore, systematic monitoring of the quality and safety of plant raw materials is essential to minimize the health risks associated with their consumption [45].

Mercury uptake by plants increases at soil pH values below 6.5 and decreases at pH values above 7.5, indicating that elevated soil pH limits the uptake of this element [46]. Plant roots can readily absorb free mercury ions from the soil, as demonstrated in experiments involving the exogenous addition of Hg^2+^. However, soils contain substantial amounts of dissolved organic matter (DOM), and the thiol and carboxyl functional groups present in DOM can strongly bind Hg^2+^, forming Hg-DOM complexes that differ in their bioavailability. Although some mechanisms involved in mercury uptake by plants have been identified, the processes governing mercury uptake from the environment still require further investigation [47].

The availability of mercury to plants in the soils analyzed by Różański et al. [48] was very low, averaging only 2.45% of the total mercury content. Mercury availability was primarily controlled by soil texture, organic matter content, and soil pH. Considering the low proportion of mobile mercury, the risk of its migration into deeper soil layers and groundwater is relatively low. Moreover, even in soils with comparatively high total mercury concentrations, its availability to plants remains very limited. Only a substantial decrease in soil pH combined with intensified mineralization of organic matter, which binds most of the mercury present in these soils (up to 73.7%), may significantly increase the mobility and/or toxicity of this metal [48].

Mercury concentrations have also been investigated in the Spiš region of Slovakia, close to an abandoned metal ore mine [49]. In that study, the highest Hg concentration in dandelion was measured in leaves (10.61 µg/g; 10,610 µg/kg), followed by stems (6.25 µg/g), while root concentrations were below 1 µg/g. For common nettle leaves, the Hg concentration reached 100 µg/kg [49]. These values are several hundred times higher than those obtained in the present study. Notably, the lowest Hg concentration in the Slovak study was found in roots, in contrast to the aerial parts of the plant. This observation suggests that Hg accumulation occurred predominantly in above-ground tissues and that the primary sources of contamination were atmospheric deposition, airborne dust, and precipitation.

This pattern was the opposite of that observed in the present study, where the highest Hg concentrations in self-collected samples of dandelion and common nettle were detected in roots. In the study by Kimáková et al. [49], Hg concentrations were not determined in flowers, which may indicate that the plants were collected at a later developmental stage after a longer growth period. In contrast, the dandelion samples analyzed in the present study were collected during the flowering season in spring, between late April and early May.

Studies involving multiple medicinal plant species have also been conducted in Ukraine, particularly in Kyiv and the Obukhiv district, areas characterized by a high degree of anthropogenic pollution [50]. Dandelion samples collected near a power plant contained Hg concentrations ranging from 4 to 6 µg/kg. Even higher concentrations (5.1–9.2 µg/kg) were detected in plants collected from a waste disposal site. The third sampling location in the study was a landscape park, where the Hg concentration in dandelion reached 5.2 µg/kg. The Hg levels reported by Ryzhenko et al. [49] were higher than those observed in commercially available herbal teas analyzed in the present study but lower than the concentrations found in self-collected plant materials.

The effects of Hg on plants have been observed as early as the seed germination stage. Such investigations were conducted in vitro using dandelion seeds and other plant species [51]. Mercury at a concentration of 0.6 mg/L reduced the germination capacity of dandelion seeds by 73.5% compared with the control group. At higher Hg concentrations (2.0 mg/L and 4.0 mg/L), seed germination was completely inhibited. The mechanisms underlying Hg-induced inhibition of seed germination and embryo development remain insufficiently understood. It has been suggested that Hg may bind to and replace sulfhydryl (-SH) groups in proteins. The formation of S-Hg-S bonds may interfere with the activity of enzymes such as amylases and proteases, which are essential for seed germination. Moreover, Hg exposure may cause seed damage, physiological disturbances, and the generation of reactive oxygen species [51].

In a large-scale study conducted in Germany, Gasser et al. [52] analyzed more than 7000 samples of herbs and medicinal plants and confirmed that none exceeded the permissible Hg limit of 0.1 mg/kg. In the present study, Hg concentrations were evaluated against a substantially lower regulatory threshold of 20 µg/kg (0.02 mg/kg) [42]. Of the 285 analyzed samples, 91 exceeded this limit, representing 32% of all samples examined.

Dolan et al. [53] investigated Hg concentrations in plant-based dietary supplements purchased in Washington State, USA. Hg concentrations ranged from 80 to 16,800 µg/kg. In supplements containing nettle leaf extract, the Hg concentration was reported to be 80 µg/kg [53].

In a Polish study conducted by Socha et al. [54] on plant-based dietary supplements, the total Hg concentration in preparations containing wild rose, along with other ingredients such as grapefruit extract, garlic, blackcurrant, and citrus bioflavonoids, ranged from 0.70 to 44.16 µg/kg, with a mean value of 9.62 µg/kg. In the same study, supplements intended to support skin, hair, and nail health that contained nettle, together with horsetail, green tea, and borage oil, showed a mean Hg concentration of 3.25 µg/kg (range: 0.22–7.91 µg/kg) [54].

Compared with these findings, the present study demonstrated the opposite trend. Hg concentrations in wild rose fruits were substantially lower (0.59 µg/kg) than those observed in common nettle samples (35.40 µg/kg).

Research conducted in northern Poland confirmed that Hg concentrations in medicinal plants may vary depending on the season of collection [41]. Statistically significant differences were observed between plant materials harvested in spring and those collected in autumn. Higher Hg concentrations were detected in plants gathered during autumn, which may be attributed to a longer growth period and prolonged exposure to environmental contamination. In that study, Hg concentrations in medicinal plants ranged from 3.6 to 82 µg/kg [41]. In contrast, the ranges observed in the present study were substantially broader: 0.60–202.83 µg/kg for common nettle, 0.28–115.06 µg/kg for dandelion, 1.26–53.68 µg/kg for silver birch, 0.97–38.92 µg/kg for common hawthorn, and 0.19–1.41 µg/kg for wild rose.

Kim and Kim [55], in their investigation of herbal medicinal products, reported a mean Hg concentration of 6.0 µg/kg and a maximum concentration of 83.6 µg/kg. All measured values were below the limits specified in the Korean Pharmacopoeia.

In contrast, Đurović et al. [56] did not detect Hg in common nettle samples collected in Serbia. Similarly, in a study conducted by Schulzki et al. [57], Hg concentrations in wild rose fruits were below the limit of quantification (LOQ) of 10 µg/kg.

A study conducted in Germany by Gasser et al. [52] on herbal medicinal products containing nettle demonstrated Hg concentrations ranging from 20 to 170 µg/kg in nettle leaves and from 20 to 210 µg/kg in nettle roots. The results obtained in the present study were highly comparable, with concentrations ranging from 14.17 to 54.00 µg/kg in leaves and from 11.54 to 202.83 µg/kg in roots. Consistent with the findings of Gasser et al. [52], Hg concentrations were higher in roots (Me = 12.70 µg/kg) than in nettle leaves (Me = 8.27 µg/kg).

Jeannin et al. [58], in a study conducted in France, reported Hg concentrations in nettle leaves below the maximum permissible levels established for agricultural crops (0.2 mg/kg). The authors observed that even in soils contaminated with Hg at levels of approximately 6 mg/kg dry weight, the translocation of Hg to nettle tissues remained limited, as Hg concentrations in leaves did not exceed 1 µg/kg dry weight. Furthermore, Hg concentrations in nettle fibers were below the limit of quantification [58].

In our previous study [59], the mean Hg concentration in common nettle was 13 µg/kg, with values ranging from 10 to 22 µg/kg. Compared with other investigated plant species, only horsetail exhibited a higher Hg concentration (14 µg/kg) than nettle.

Kowalska [45] reported Hg concentrations below the limit of quantification (LOQ = 5 µg/kg) in dandelion herb as well as in hawthorn and rosehip fruits.

García-Rico et al. [60], in a study of plant-based dietary supplements available in Mexico and originating from Mexico, the United States, and Korea, demonstrated that Hg concentrations in multicomponent preparations containing common hawthorn and nettle were below 0.24 µg/g. These values were considerably lower than those observed in the present study.

In research conducted in Eastern Anatolia, Turkey, Levent et al. [61] found that concentrations of the investigated heavy metals (excluding Hg) in *Rosa canina* L. were comparable to those reported for samples collected in other regions of the world.

A study conducted in Romania on commonly used medicinal plants, including silver birch, showed that Hg concentrations exceeded regulatory threshold values in 28% of the analyzed samples. However, Hg was not detected in the birch samples included in that study [62].

Research carried out in Alaska, USA, included dwarf birch (*Betula nana*), for which Hg concentrations of approximately 8 µg/kg were reported in the leaves [63]. These values were substantially lower than those observed in the present study, where the mean concentration in silver birch leaves was approximately 22 µg/kg. A Canadian study investigating yellow birch (*Betula alleghaniensis*) reported a leaf Hg concentration of 12 µg/kg [64]. Compared with silver birch, this value was lower than, but relatively close to, the median Hg concentration observed in the present study (approximately 15 µg/kg).

In Norway, Hg concentrations in moor birch (*Betula pubescens*) ranged from 11 to 28 µg/kg [65]. When compared with the concentrations measured in silver birch in the present study (approximately 4–54 µg/kg), these values appear broadly comparable. Forest ecosystems are known to play a crucial role as sinks for atmospheric Hg, largely because of the extensive leaf surface area available for the interception and accumulation of Hg. Within forest ecosystems, trees are second only to soils in terms of Hg storage capacity [66]. The present findings support this observation, demonstrating significantly higher Hg concentrations in birch leaves than in buds. The duration of tissue development may also contribute to these differences, as buds persist for only a relatively short period during the growing season.

Medicinal plants are most commonly consumed as aqueous infusions rather than as dried raw material itself. Therefore, the assessment of consumer exposure should take into account the transfer of Hg from plant tissues into the prepared beverage. Available evidence suggests that only a small fraction of the total mercury present in plant material is extracted into the aqueous phase during infusion preparation, with the extraction efficiency being influenced by the chemical speciation of mercury, the physicochemical properties of the plant matrix, and brewing conditions [67]. Studies investigating tea infusions have demonstrated that mercury concentrations in the final beverage are generally considerably lower than those determined in the corresponding dried leaves, indicating limited leaching of this element into water. For instance, Árvay et al. [67] reported mercury concentrations of only 0.03–0.05 μg/L in tea infusions despite measurable mercury levels in the respective dried plant material. Consequently, the determination of total mercury concentrations in dried herbal samples may overestimate the actual dietary exposure associated with the consumption of herbal infusions. Nevertheless, the extent of mercury transfer is known to vary among plant species, and data for many medicinal herbs remain scarce. Therefore, a more accurate assessment of consumer exposure would require direct measurements of mercury concentrations in prepared infusions. Furthermore, the mean THQ and HI values for tea infusions were below 1, indicating no health risk associated with metal exposure through their consumption [68].

The studies discussed above clearly indicate that Hg concentrations in plant-derived products exhibit substantial variability, ranging from levels below analytical detection limits to concentrations exceeding permissible regulatory thresholds. It should also be noted that herbal medicines and dietary supplements frequently consist of multiple ingredients collected from different sources and at different times, contributing to considerable variability in contaminant concentrations [44,69].

A limitation of the present study is the relatively small number of commercial product samples and plant species included in the investigation. This was primarily due to difficulties in procurement, as the availability of certain herbal products on the Polish market is limited. Additionally, soil samples from the locations where the plants were collected were not analyzed, which may have limited the interpretation of the results.

## 3. Materials and Methods

### 3.1. Materials

The investigated plant material consisted of raw materials obtained from five medicinal plant species: wild rose (*Rosa canina* L., fruits), common hawthorn (*Crataegus monogyna* L., fruits and flowers), silver birch (*Betula pendula*, leaves and buds), dandelion (*Taraxacum officinale*, herbs, roots, and flowers), and common nettle (*Urtica dioica* L., roots and leaves). A total of 285 samples were included in the study. The detailed number of samples collected from each plant species is presented in Table 3.

Commercial products—samples (N = 56)—were purchased from herbal stores, pharmacies, drugstores, and online retailers. The products originated from various manufacturers. According to the manufacturers’ declarations, the herbal materials were sourced in Poland, either from contract cultivation or from wild-harvested plant populations. All herbal tea samples were in the form of dried plant material.

**Figure 5:**
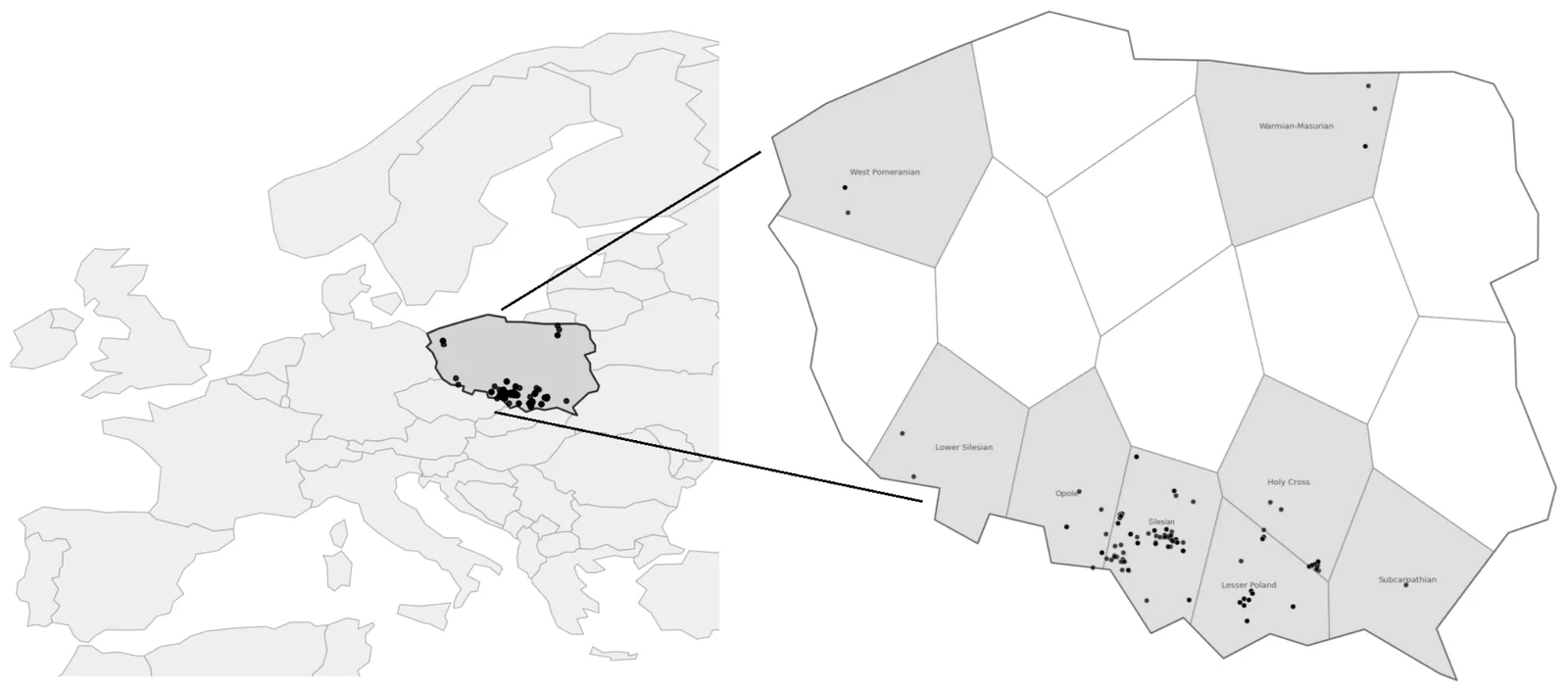
Collection sites of wild populations of medicinal plants in Poland, (it was prepared using Copilot).

Self-collected plant samples (N = 229) were gathered between 2022 and 2024. The plants were collected from natural habitats located in eight voivodeships (provinces) of Poland: Silesian, Lower Silesian, Lesser Poland, Subcarpathian, West Pomeranian, Świętokrzyskie (Holy Cross), Opole, and Warmian–Masurian (Figure 5). Sampling locations were selected randomly. To minimize the potential impact of local contamination, all collection sites were situated at least 100 m away from roads and agricultural fields. In the case of common nettle and dandelion, whole plants were harvested and subsequently separated into their morphological parts prior to drying. For common hawthorn and wild rose fruits, the pulp was separated from the seeds before further processing. Silver birch samples did not require any additional preparation. After collection, all plant materials were dried in a shaded, dry, and well-ventilated place for approximately 30 days until a constant dry mass was obtained.

Approximately 10 g of dried plant material was used to prepare a representative sample. The samples were crushed using a porcelain mortar. Plant materials with higher hardness were additionally homogenized using an Analytical Mill A11 basic (IKA, Warsaw, Poland) to ensure uniform sample preparation. Subsequently, the samples were stored in resealable bags made of durable low-density polyethylene (LDPE) film. Subsamples weighing approximately 55 mg were then prepared using an analytical balance (OHAUS Pioneer, OHAUS Europe GmbH, Nänikon, Switzerland) and subjected to further analysis.

### 3.2. Analytical Method

Hg content was determined using an AMA 254 atomic absorption spectrometer (Altec, Prague, Czech Republic). This instrument enables the measurement of total Hg regardless of its chemical form. The analytical technique is based on the ability of Hg to be released from its compounds and converted into atomic form. This process is applicable to both organic and inorganic Hg-containing compounds. The AMA 254 analyzer performs Hg determination in three stages. In the first stage (decomposition), the sample is placed in a combustion tube and heated to approximately 750 °C, resulting in the thermal decomposition of Hg compounds and the release of Hg in the gaseous state. During the second stage (collection), Hg vapor, carried by an oxygen stream, passes through a catalytic column and is trapped in an amalgamator. After all Hg has been collected, the amalgamator is heated to approximately 900 °C, releasing the accumulated Hg vapor into the detection system. The third stage (detection) involves directing the released Hg vapor into a dual-path optical cuvette system. One optical path is optimized for the determination of low Hg concentrations, while the second is designed for higher Hg concentrations. This dual-cuvette system extends the analytical range and enables the accurate determination of Hg over a broad concentration spectrum.

The atomic absorption spectrometer is equipped with a Hg lamp emitting radiation at a wavelength of 253.7 nm and a silicon UV photodiode detector used for the quantification of Hg. The limit of detection (LOD) was 0.01 ng (0.00001 µg), as specified by the manufacturer [70,71]. The limit of quantification (LOQ) was determined from ten replicate measurements of blank samples and was found to be 0.10 μg/kg. Taking into account the sample masses used for the determination of mercury in medicinal plants (mean sample weight: 55 mg), the limit of quantification (LOQ) was 0.18 µg/kg. The following measuring conditions were used: wavelength 253.65 nm, carrier gas, oxygen (O_2_ purity ≥ 99.5%); inlet pressure, 200–250 kPa. The duration of each analytical step was as follows (in seconds): drying, 60; decomposition, 150; measurement, 45. Prior to each measurement, the apparatus was cleaned with air and distilled water in accordance with the analytical procedure. The instrument was used with its original factory calibration. Certified reference material (CRM) was used to ensure quality control of the method. Mixed Polish Herbs (INCT-MPH-2, Institute of Nuclear Chemistry and Technology Department of Analytical Chemistry, Warsaw) was analyzed; the mean result of six determinations was 0.0165 ± 0.0019 mg/kg, with a recovery of 95.93%. The obtained recovery rate indicates the satisfactory accuracy of the applied analytical procedure. The instrument was controlled by an external PC running Windows^®^, with software enabling, among other functions, the creation of calibration curves, statistical analysis of the results, and monitoring of the ongoing process with a real-time signal display. Prior to each analysis, the device was cleaned in air and then in deionized water (Elix Essential Water Purification, Merck, Darmstadt, Germany). Three measurements of each sample were performed, and their arithmetic mean was calculated.

### 3.3. Statistical Analysis

The obtained results were statistically analyzed using Microsoft Excel 2007 and Statistica ver. 13.3 PL for the Windows operating system [72]. The following were determined: arithmetic mean (AM), standard deviation (SD), median (Me), first quartile (Q1), third quartile (Q3), and coefficient of variation (CV). The results are given to two decimal places. Non-parametric tests were used to compare the tested sample groups: the Mann–Whitney U test (for two groups) and the Kruskal–Wallis ANOVA by ranks (for more than two groups). Differences were considered statistically significant at *p* < 0.05.

## 4. Conclusions

Although self-collection of medicinal plant materials, including common nettle (*Urtica dioica* L.), dandelion (*Taraxacum officinale*), silver birch (*Betula pendula*), common hawthorn (*Crataegus monogyna* L.), and wild rose (*Rosa canina* L.), may be attractive owing to the well-documented therapeutic properties of these plants, it may involve a risk of Hg contamination. The present study demonstrated that more than 38% of self-collected plant samples exceeded the permissible Hg concentration limit. Therefore, the collection and consumption of wild medicinal plants may represent an additional source of human Hg exposure. In contrast, only 5% of the analyzed commercial products exceeded the regulatory threshold, suggesting that commercial products are generally safer for consumption.

The highest Hg concentrations were observed in the roots of self-collected medicinal plants, indicating that this morphological plant part may pose the greatest risk with respect to Hg accumulation.

It should be emphasized that human exposure to mercury may result from multiple environmental pathways, including dietary intake, drinking water consumption, and inhalation. Therefore, the cumulative contribution of these sources should be considered when assessing the overall exposure to this contaminant.

Given the continuously increasing demand for and market value of herbal medicines, ensuring the safety, quality, and efficacy of medicinal plants and herbal products remains of critical importance. Regular monitoring of toxic element concentrations in plant materials used in phytotherapy is therefore fully justified and necessary.

## Figures and Tables

**Table 1:** Statistical characteristics of mercury (Hg) content in the investigated medicinal plants, taking into account individual species [µg/kg].

Plant	N	AM ± SD	Me	Min	Max	Quartiles		CV [%]
						Q1	Q3	
all	285	18.68 ± 22.48	13.14	0.19	202.83	5.54	24.18	120
Wild rose	24	0.67 ± 0.36	0.61	0.19	1.41	0.33	0.94	54
Common hawthorn	30	7.93 ± 7.03	5.91	0.97	38.92	4.04	10.60	89
Dandelion	97	19.08 ± 20.76	13.00	0.28	115.06	5.66	23.59	109
Common nettle	71	30.91 ± 31.50	23.80	0.60	202.83	18.33	32.45	102
Silver birch	63	16.27 ± 12.07	12.68	1.26	53.68	7.96	18.10	74

N—number of samples, AM—arithmetic mean, SD—standard deviation, Me—Median, Min—Minimum, Max—Maximum, Q1—first quartile, Q3—third quartile, CV—coefficient of variation.

**Table 2:** Statistical analysis of mercury (Hg) content according to plant species, morphological part, and origin (commercial products vs. wild populations) [µg/kg].

	Morphological Part	N	AM ± SD	Me	Min	Max	Quartiles		CV [%]
							Q1	Q3	
COMMERCIAL PRODUCTS									
Wild rose	fruits	6	0.92 ± 0.41	0.98	0.19	1.34	0.81	1.24	45
Common hawthorn	flowers	4	11.84 ± 1.20	11.81	10.60	13.14	10.84	12.84	10
	fruits	9	5.11 ± 2.72	5.75	0.97	9.82	3.26	6.67	53
Dandelion	roots	5	2.63 ± 1.98	3.75	0.28	4.62	0.74	3.78	75
	flowers	4	3.79 ± 2.27	4.10	0.87	6.09	2.06	5.52	60
	herbs	4	6.01 ± 4.66	3.76	3.50	13.00	3.62	8.39	78
Common nettle	roots	4	10.13 ± 6.44	12.70	0.60	14.54	6.33	13.94	64
	leaves	9	11.22 ± 8.27	8.27	3.13	24.99	4.47	17.99	74
Silver birch	leaves	5	10.16 ± 9.48	5.54	2.08	23.29	2.87	17.04	93
	buds	6	4.53 ± 3.31	4.49	1.26	7.96	1.63	7.39	73
WILD POPULATION									
Wild rose	fruits	18	0.59 ± 0.32	0.47	0.24	1.41	0.33	0.80	54
Common hawthorn	fruits	17	8.49 ± 8.77	5.40	2.14	38.92	4.15	10.52	103
Dandelion	roots	28	28.47 ± 23.10	21.82	4.96	102.34	13.57	32.01	81
	flowers	28	8.00 ± 4.79	6.50	3.42	26.00	4.60	10.33	60
	herbs	28	27.78 ± 23.78	19.14	6.76	115.06	14.81	31.48	86
Common nettle	roots	29	46.89 ± 43.53	32.57	11.54	202.83	22.29	50.66	93
	leaves	29	23.90 ± 7.75	21.47	14.17	54.00	18.56	28.12	32
Silver birch	leaves	29	22.36 ± 14.78	14.93	4.18	53.68	10.67	33.76	66
	buds	23	12.98 ± 2.71	12.68	7.82	18.10	10.78	14.75	21

N—number of samples, AM—arithmetic mean, SD—standard deviation, Me—Median, Min—Minimum, Max—Maximum, Q1—first quartile, Q3—third quartile, CV—coefficient of variation.

**Table 3:** Number of herb and herbal tea samples used in the study.

	Wild Rose *Rosa canina* L.		Common Hawthorn *Crataegus monogyna* L.		Dandelion *Taraxacum officinale*		Common Nettle *Urtica dioica* L.		Silver Birch *Betula pendula*	
morphological part	commercial products	wild population	commercial products	wild population	commercial products	wild population	commercial products	wild population	commercial products	wild population
leaves	-	-	-	-	5	29	-	-	9	29
buds	-	-	-	-	6	23	-	-	-	-
fruits	6	18	9	17	-	-	-	-	-	-
flowers	-	-	4	-	-	-	4	28	-	-
roots	-	-	-	-	-	-	5	28	4	29
herbs	-	-	-	-	-	-	4	28	-	

## Data Availability

The data presented in this study are available on request from the corresponding author.
